# Dietary Modulation of Intestinal Microbiota: Future Opportunities in Experimental Autoimmune Encephalomyelitis and Multiple Sclerosis

**DOI:** 10.3389/fmicb.2019.00740

**Published:** 2019-04-16

**Authors:** Yuying Fan, Junmei Zhang

**Affiliations:** Department of Pediatrics, Shengjing Hospital of China Medical University, Shenyang, China

**Keywords:** multiple sclerosis, experimental autoimmune encephalomyelitis, microbiota, dietary habits, short-chain fatty acid, microRNA

## Abstract

Multiple sclerosis (MS) is an autoimmune disease that affects the functioning of the central nervous system (CNS). Recent studies on MS and its animal model, experimental autoimmune encephalomyelitis (EAE), have shown that the composition and abundance of microbes in the intestinal microbiota are an environmental risk factor for the development of MS and EAE. Changes in certain microbial populations in the gastrointestinal tract can cause MS in humans, but MS inflammation can be reduced or even prevented by introducing other commensal microbes that produce beneficial metabolites. Other risk factors for MS include the presence of an altered gut physiology and the interaction between the intestinal microbiota and the immune system. Metabolites including short-chain fatty acids (SCFAs), such as butyrate, are the primary signaling molecules produced by the intestinal microbiota that interact with the host immune system, suggesting an association between MS pathophysiology and gut microbiota. In addition, several host microRNAs present in the gut have been found to interact with the intestinal microbial community, these interactions may indirectly affect the neurological system. Increasing evidence has shown that regulation of the intestinal microbiota is an important approach for reducing MS inflammation. Thus, here we review the use of diet to alter the gut microbiota and its application in the treatment and prevention of MS.

## Introduction

Multiple sclerosis (MS) is a chronic autoimmune disease that causes demyelination and degeneration in the central nervous system (CNS) and is characterized by inflammation and white matter lesions. These lesions consist of CNS cells, such as astrocytes and microglia, along with activated immune cells. The most popular method of studying MS is to use the animal model of experimental autoimmune encephalomyelitis (EAE) (Berer et al., 2011; Lee et al., 2011). More research is needed to understand how an etiological agent might trigger MS and/or EAE.

Certain genetic constitutions increase the susceptibility to MS, and these genetic factors may interact with environmental factors to cause MS (Ascherio, 2013; Koch et al., 2013). Environmental factors include dietary habits (Riccio and Rossano, 2015; Haghikia and Linker, 2018), which can alter the intestinal microbial community, and thus there is considerable variability among individuals. Dietary habits are the primary determinant of the microbial composition and function in the gut (Tilg and Moschen, 2015; Martinez et al., 2016) and thus greatly responsible for shaping the microbial structure. Studies have suggested that the presence of certain gut microbiomes could promote or prevent MS development (Jangi et al., 2016; Liu et al., 2016; Rothhammer and Quintana, 2016). For example, certain bacterial species, such as those of the genus *Bifidobacterium* and the phylum Firmicutes, are associated with good health, whereas those of the phylum Bacteroidetes have been linked to disease development (Johnson et al., 2017; Azad et al., 2018; Hiippala et al., 2018). Therefore, it would be beneficial to explore how dietary modifications can be used to mitigate the effects of MS. Individualized nutrition plans can be used to restore desirable gut flora populations to reverse microbial dysbiosis, thereby helping to prevent or treat MS. In this review, we explore how the intestinal microbiota affects the pathophysiology of MS and EAE and how this knowledge could be applied to develop therapies for MS treatment.

## Interactions Between the Intestinal Microbiota and a Normal CNS

In humans, the gut microbiota plays a crucial role in regulating physiological processes such as metabolism and immunity. They can also affect brain functions through interactions with the immune, nervous and endocrine systems (Crane et al., 2015; Yano et al., 2015). The metabolites of gut microbiota have the potential to prevent inflammation via their interactions with the CNS. One study confirmed the presence of a relationship between the intestinal mucosa and the brain by demonstrating that polysaccharide A (PSA) from *Bacillus fragilis* mediate microflora migration (Ochoa-Reparaz et al., 2010; Wang et al., 2014b). This finding suggests the presence of a gut–microbiota–brain axis in the human body. This axis is thought to control the biochemical communication between the CNS and the enteric nervous system (ENS) of the gastrointestinal (GI) tract that appears to be mediated by the intestinal microbiota (Gareau, 2014). The ENS communicates with the CNS via both afferent and efferent pathways of the autonomic nervous system. The afferent pathway communicates signals from the GI tract to the CNS, whereas the efferent pathway communicates signals from the CNS to the GI tract. The vagus nerves are involved in the afferent functions and can recognize the presence of microbial products and cell wall components, and efferent neural signals can affect GI motility, secretion and epithelial permeability. The effects of such efferent signals alter the composition of the intestinal microbiota by changing the physical environment (Bjelobaba et al., 2017; In’t Veld et al., 2017).

Changes in the gut microbiota composition can also result from the release of glucocorticoids, mineralocorticoids, or catecholamines by the pituitary and adrenal glands, which are regulated by the hypothalamus. This release can also increase the gut epithelial permeability and immune responses (Bellavance and Rivest, 2014; Yin et al., 2018). Furthermore, the gut microbiota can alter cytokine secretion, thereby regulating neurotransmitter release in the central and peripheral nervous systems. The regulation of neurotransmitter release and other interactions between gut bacteria and the host can lead to the production of biogenic amines and neuroactive substances, such as γ-aminobutyric acid (GABA), dopamine and serotonin, with immunoregulatory effect from host cells (Wong et al., 2015). These neuroactive amines can affect important processes within the body including the digestive, immune, and nervous systems, thereby aiding the maintenance of homeostasis (Rodriguez et al., 2015).

The intestinal microbiota can influence not only the function but also the development of the host immune system (Belkaid and Harrison, 2017). Different subsets of cells in the immune system are affected by different microbiomes. In particular, the gut microbiota plays an important role in the fermentation process, converting indigestible carbohydrates into acetate, propionate and butyrate – the three primary SCFAs. SCFAs are known to decrease inflammation and inhibit the production of histone deacetylase, and recent evidence suggests that SCFAs are instrumental in neuroimmune homeostasis (Erny et al., 2015). Notably, SCFAs can activate the brain’s immune response to inhibit histone deacetylase through epigenetic mechanisms. This inhibition then induces regulatory T cell (Treg cell) production in the intestine. Treg cells play a role in maintaining the blood–brain barrier (BBB) and regulating the activity of CNS microglia by simultaneously limiting the microglial size and effect in the brain while also activating them as needed (Erny et al., 2015; Sampson et al., 2016). This suggests that SCFAs can affect CNS homeostasis and maturation. In addition, SCFAs are considered anti-inflammatory molecules as they induce cytokine production and interact with the G-protein coupled receptor 45. The intestinal microbiota can release immune antigens, such as peptidoglycan and PSA, that induce immune responses and thus regulate brain function (Desbonnet et al., 2015). SCFA production varies based on the gut microbiota composition, which, in turn, is affected by the type and amount of dietary fiber consumed. The gut microbiota also facilitates amino acid metabolism in the digestive system. Building upon these findings, more studies are investigating how microbial tryptophan metabolites affect immune and neurological processes by functioning as mediators in the human body (Desbonnet et al., 2015).

## The Role of the Intestinal Microbiota in MS Pathophysiology

Multiple sclerosis is a heterogenous neurological disease that is mediated by the immune system and is attributable to both genetic and environmental factors. It is considered that MS onset is triggered in genetically susceptible individuals by environmental factors (Belbasis et al., 2015), such as disturbed gut microbiota. A compromised immune system due to alterations in gut microbial composition (e.g., intestinal dysbiosis) can trigger MS development or aggravate its effects. Studies comparing age- and gender-matched groups of MS patients and healthy individuals demonstrated that the gut microbiomes in MS patients were significantly different from those in healthy individuals (Chen et al., 2016). The study reported an increased abundance of *Pseudomonas*, *Mycoplana*, *Haemophilus*, *Blautia*, and *Dorea* microbes and a decreased abundance of *Parabacteroides*, *Adlercreutzia*, and *Prevotella* microbes in MS patients (Chen et al., 2016).

### Intestinal Dysbiosis and EAE

Studies using the animal model of MS, EAE, have reported strong evidence suggesting a link between the gut microbiota and MS development. One study showed that germ-free mice and antibiotic-treated mice had a significantly lower rate of EAE than specific pathogen-free mice (Kennedy et al., 2018). The intestinal microbiota has been shown to play a vital role in maintaining the balance between inflammatory and anti-inflammatory responses of the immune system during EAE development. It also plays a critical role in regulating the BBB permeability, limiting astrocyte pathogenicity, activating microglia and expressing myelin genes (Hoban et al., 2016; Rothhammer et al., 2016).

One study on non-obese diabetic mice showed that the intestinal microbiota could affect EAE development and severity. In antibiotic-treated mice, EAE development was delayed and disease progression was stalled, thereby reducing the disease severity (Colpitts et al., 2017). Another group of mice developed a second form of EAE that was more severe than the first form. These mice, but not those with the first form of EAE, exhibited intestinal dysbiosis, and this difference could be observed from an early stage of disease progression (Colpitts et al., 2017). In addition, the commensal microbiota facilitated the responses of inflammatory T helper (Th) cells, such as Th1 and Th17, which prevent future infections. These Th cells could be recruited by B cells and activated by dendritic cells in EAE. These findings indicate that the commensal microbiota are involved in the prevention of EAE exacerbation in diseased mice (Wang et al., 2014a).

Building on these findings of the role of the gut microbiota in EAE onset and severity, several studies have examined how commensal microbes and their products affect the disease. *B. fragilis*, a gram-negative anaerobe and universal gut microbe, produces PSA on its surface. Pure PSA administration has been found to decrease EAE severity (Ochoa-Reparaz et al., 2010; Wang et al., 2014b), delay the disease onset and decrease its cumulative score, suggesting that it plays a crucial role in protection against EAE. Butyrate is a microbial metabolite of particular interest because it is produced in the gut and has shown to increase the effectiveness and production of circulating Treg cells in mice (Haghikia et al., 2015). The effect of butyrate is important as Treg cells play a crucial role in the development of peripheral tolerance, which prevents the onset of autoimmune diseases. Treg cells can differentiate into anti-inflammatory and pro-inflammatory Treg cells. In one study, anti-inflammatory Treg cell levels could be enhanced in mice by SCFA administration, which suppressed the production of pro-inflammatory Treg cells (Haghikia et al., 2015; Chitrala et al., 2017). To further investigate this observation, Haghikia et al. (2015) studied the effects of dietary fatty acids of different lengths on the immune system and consequently on EAE pathophysiology in mice. They found that fatty acids with shorter chains were more effective at regulating and reducing EAE symptoms, thereby decreasing the disease severity. This effect could be promoted by feeding the mice with a diet richer in fiber than normal chow, which increased the propionate and fecal acetate contents responsible for this effect. A positive correlation has been reported between butyrate levels and FoxP3+ Treg cells present in the lymph nodes and spleen of mice. Notably, butyrate can also significantly decrease the production of interferon gamma (IFN-γ), a Th1 cytokine, and increase that of interleukin 17, another Th1 cytokine.

### Intestinal Dysbiosis and MS

**FIGURE 1:**
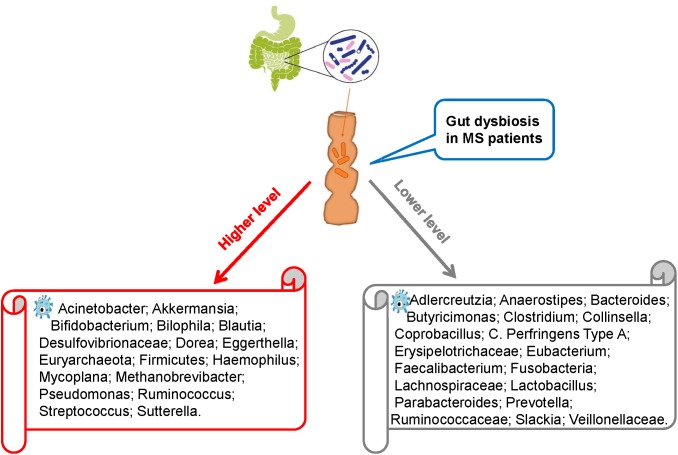
Intestinal dysbiosis in MS patients.

Recent studies comparing intestinal microbiota present in the feces of MS patients and healthy individuals have revealed intestinal dysbiosis in MS patients, in whom certain microbial populations such as *Pseudomonas*, *Mycoplana*, *Haemophilus*, *Blautia*, *Dorea*, *Pedobacter*, and *Flavobacterium* were enriched and others such as *Prevotella*, *Parabacteroides*, *Adlercreutzia*, *Collinsella*, *Lactobacillus*, *Coprobacillus*, and *Haemophilus* were depleted compared with those in healthy controls (Figure 1; Chen et al., 2016; Tremlett et al., 2016b). Similarly, Miyake et al. conducted a longitudinal study to compare the intestinal microbiota of Japanese patients with relapsing-remitting MS (RRMS) with healthy Japanese people (control). Compared with the control group, the RRMS group demonstrated a moderate level of dysbiosis and significant changes in the abundance of 21 microbial species. However, the bacterial diversity in MS patients remained similar to that in the control patients, which is an interesting finding because the bacterial diversity is known to decrease in other diseases such as inflammatory bowel disorders. To further evaluate the bacterial diversity, Schirmer et al. (2016) examined the effect of *Dorea* species in the body and suggested that certain *Dorea* species promote inflammation by facilitating IFN-γ production, metabolizing sialic acids and degrading mucin. *Dorea* populations have been suggested to be linked to MS development as MS patients exhibit increased *Dorea* abundance. This finding suggests that *Dorea* microbes plays pro-inflammatory roles. Notably, *Dorea* species, similar to T cells, can exhibit both pro-inflammatory and anti-inflammatory properties, the selection between which could be guided by environmental factors, such as the surrounding gut microbiota or nutritional composition.

Research has shown that MS patients exhibit decreased *Faecalibacterium* abundance compared with healthy individuals (Machiels et al., 2014). This decrease has been found to be attributable to glatiramer acetate administration used for treatment in MS patients. This treatment also decreases the abundance of *Bacteroidaceae*, *Ruminococcus*, *Lactobacillaceae*, *Clostridium*, and other Clostridiales microbes. To further investigate this effect, Tremlett performed three experiments on children with MS and found that their gut microbiota were significantly different from that of healthy children (Tremlett et al., 2016a; McKay et al., 2017; Tremlett and Waubant, 2018). Although the abundances of Firmicutes, Archaea, Euryarchaeota, and Proteobacteria (Desulfovibrionaceae) microbes were increased in children with MS, those of *Lachnospira* (Lachnospiraceae), Verrucomicrobia (Ruminococcaceae), and Fusobacteria microbes were decreased. Furthermore, children with MS who did not exhibit Fusobacteria population in the gut were more likely to relapse, indicating a possible link between Fusobacteria abundance and MS relapse (Tremlett et al., 2016c). Although the study by Tremlett showed no variations in immune markers between children with and without MS, it showed a negative correlation between Bacteroidetes abundance and Th17 level in the MS group and a positive correlation between Fusobacteria abundance and the Treg cell population in the control group.

Branton et al. (2016) examined brain biopsy specimens of 23 MS patients and 21 non-MS patients with other diseases and found that compared with the MS patients, non-MS patients exhibited a greater diversity of bacterial RNA in the cerebral white matter but no significant variations in the gut bacterial diversity. However, the gut microbial structures were different between MS and non-MS patients. The MS patients were also likely to exhibit decreased abundance of Bacteroidetes and Firmicutes microbes in the gut compared with non-MS patients. Notably, healthy digestive systems harbor abundant populations of Firmicutes and Bacteroidetes microbes. Thus, the evidence presented by Branton et al. suggests the presence of differences in the abundance of microbiota at sub-phylum levels between MS and non-MS patients.

An increased abundance of *Akkermansia* spp. (phylum Verrucomicrobia) in MS patients compared with that in non-MS patients has also been reported. Members of this genus degrade mucin to produce SCFAs, which, as noted above, can aid the suppression of inflammation (Jangi et al., 2016). However, *Akkermansia* may also possess a pro-inflammatory property as it can upregulate genes associated with innate and adaptive immune responses. This property can affect antigen presentation, adaptive cell adhesion molecules and T cell production. As explained above, butyrate can also regulate T cell production – an increased butyrate level has been linked to a greater Treg cell population. Thus, it is hypothesized that changes in intestinal microbiota composition by certain mechanisms could increase the risk of developing MS. To investigate this hypothesis, Cantarel et al. (2015) compared intestinal microbiota between seven RRMS patients and eight healthy individuals with vitamin D deficiency and found that compared with the healthy individuals, the RRMS patients had significantly lower *Faecalibacterium* abundance and higher *Ruminococcus* abundance. Species of these two genera are known to produce butyrate in the human body.

In summary, many studies have demonstrated that MS patients exhibit intestinal dysbiosis. Compared with healthy individuals, MS patients often have lower abundance of *Faecalibacterium*, Bacteroidaceae and *Prevotella* populations, suggesting a link between gut microbiota and MS pathophysiology. To deepen our understanding of this relationship, more large-scale longitudinal studies are needed to evaluate the effect of various endogenous and environmental factors, including age, gender, geographic location, dietary habits and genetic background, on gut microbiota and consequently MS outcome.

### Is the Gut Microbiota–MicroRNA Interaction Related to MS/EAE?

MicroRNAs (miRNAs) are increasingly used as biomarkers for several autoimmune diseases because these molecules are stable in the body and are small. In addition to their function as biomarkers, miRNAs have been suggested to affect MS pathophysiology (Jagot and Davoust, 2016). One study could identify the clinical progression of MS by examining the expression profiles of miRNAs in the body (Mancuso et al., 2015). In MS patients and EAE subjects, miRNAs have shown to mediate the upregulation of miR-29b, miR-141, miR-200a, miR-155, miR-223, miR-326, let-7e, and miR-448 expression along with a significant downregulation of miR-15a/16-1 and miR-15b expression in CD4+ T cells (Ifergan et al., 2016; Liu et al., 2017; Chen et al., 2018). In addition, miRNA has been shown to decrease the miR-20b expression and significantly increase the miR-21 and miR-590 expression in Th17 cells compared with those in Th1, Th2 and inducible Treg cells (Chen et al., 2018). This suggests that miRNAs and the gut microbiota interact to regulate disease progression in the body. Studies have suggested that miRNAs produced by host cells regulate the gut microbiota and that the gut microbiota, in turn, can trigger miRNA production in the host.

The relationships between the gut microbiota and miRNAs have been examined in liver diseases, cancers and intestinal epithelial disease. Table 1 describes the findings of relevant articles. Notably, no study has yet analyzed the effect of the gut microbiota–miRNA interaction on MS and EAE, and there is little knowledge on how gut microbiota affects brain function. Nonetheless, knowledge from studies on the gut–brain axis can be applied here as there are notable overlaps between some parts of the axis and miRNA functions. These overlaps include the immune system, hypothalamic–pituitary–adrenal (HPA) axis and vagus nerve. In addition, MS severity in humans could be limited by microbial metabolites, such as butyrate, which triggers miR-375 expression. Microbial metabolites could also have beneficial effects via the regulation of tryptophan metabolism.

**Table 1:** Studies on the interactions between gut microbiota and miRNAs in the neurological system.

Study title (year)	Main findings on gut microbiota–miRNA interactions	Related miRNA	References
Microbial regulation of hippocampal miRNA expression: Implications for transcription of kynurenine pathway enzymes (2017)	Gut microbiota regulates the expression of miRNAs, especially miR-294-5p, in the hippocampus, thereby regulating hippocampal gene expression that is critical for hippocampal development.	miR-294-5p	Moloney et al., 2017
Microbial regulation of microRNA expression in the amygdala and prefrontal cortex (2017)	Microbiota composition and activity are involved in the regulation of miRNA expression (especially miR-219a-2-3p and miR-190a-5p) in the amygdala and prefrontal cortex. This regulation also depends on the presence of functional microbiota during critical windows of neurodevelopment.	miR-219a-2-3p, miR-190a-5p	Hoban et al., 2017
A pilot study on the effects of probiotic supplementation on neuropsychological performance and microRNA-29a-c levels in antiretroviral-treated HIV-1-infected patients (2017)	HIV patients who received a probiotic supplement for 6 months demonstrated an improvement in some neurocognitive functions. This treatment was safe and did not significantly modify the miRNA levels in the cerebrospinal fluid, indicating that modifications in gut microbiota can impart specific neurological benefits in HIV-1 patients without significant side effects.	microRNA-29a-c	Ceccarelli et al., 2017
Effects of gut microbiota on the microRNA and mRNA expression in the hippocampus of mice (2017)	Gut microbiota significantly influences the expression levels of miRNAs (especially miR-190a-5p, miR-421-3p and miR-539-5pmiR-758-5p) and mRNAs in the hippocampus. The results provide original and valuable data for researchers to further investigate the microbiota–gut–brain axis.	miR-190a-5p, miR-421-3p, miR-539-5p miR-758-5p	Chen et al., 2017
Total abdominal irradiation exposure impairs cognitive function involving miR-34a-5p/BDNF axis (2017)	Total abdominal irradiation (TAI) caused cognitive impairment in mouse models and upregulated miR-34a-5p expression in the small intestine and peripheral blood, which downregulated the expression of brain-derived neurotrophic factor in the hippocampus. Intravenous injections of miR-34a-5p antagomiR in the tail inhibited the increase of miR-34a-5p level in the peripheral blood and hippocampus and recovered the cognitive dysfunction.	miR-34a-5p	Cui et al., 2017


**FIGURE 2:**
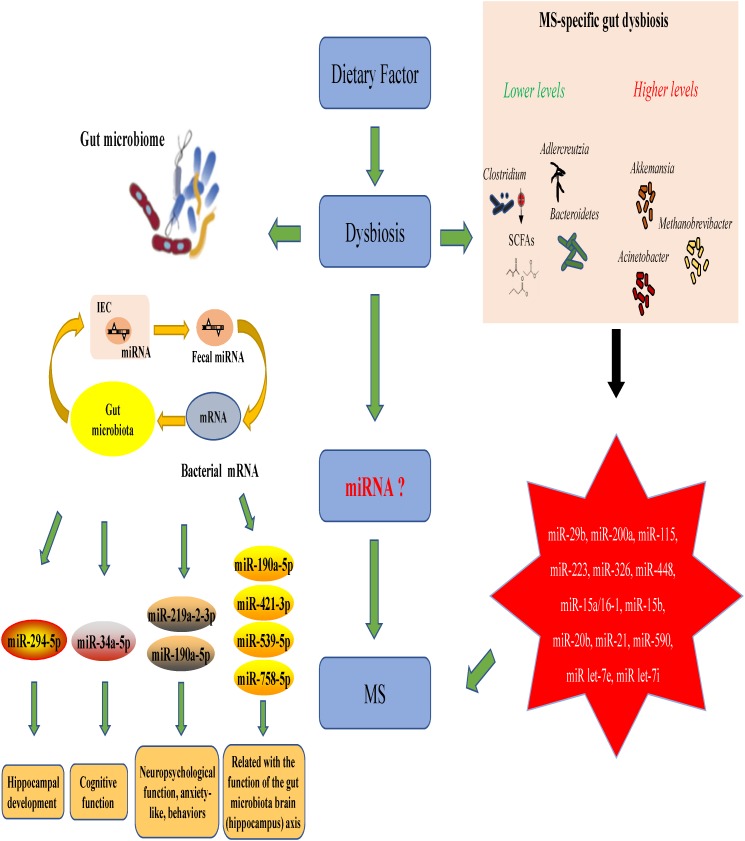
The role of gut microbiota–miRNA interactions in the onset and progression of MS/EAE.

A recent study discovered a new mechanism underlying MS pathogenesis that involves exosome miRNAs from the plasma (Kimura et al., 2018). Exosomes miRNAs, such as let-7i, circulate through the bloodstream and prevent Th1 and Th17 cells from differentiating during MS onset (Kimura et al., 2018; Tse et al., 2018). This finding suggests that the transfer of extrinsic miRNAs by exosomes is critical for the onset of autoimmune diseases. The relationships between miRNAs and gut microbes likely impact the pathophysiology of MS and EAE. The suggested mechanism is shown in Figure 2. This theoretical mechanism will need to be tested in the future.

The intestinal microbial composition varies based on dietary habits. As mentioned above, compared with healthy controls, MS patients exhibit intestinal dysbiosis with decreased abundance of *Clostridium*, Bacteroidetes and *Adlercreutzia* microbes in addition to other microbes known for their role in regulating the body’s immune responses (Miyake et al., 2015). *Clostridium* species are the major producers of SCFAs, which aid Treg cells in suppressing inflammation in the body. Fecal miRNAs also influence the gut microbial composition, which, in turn, influences the miRNAs present in intestinal epithelial cells. The effects of gut microbiota–miRNA interaction overlap with those of the gut microbiota–hippocampus axis and are related to the development of the hippocampus, cognitive function and neuropsychological functions, such as anxiety. Studies have shown a link between MS/EAE and the following miRNA molecules: miR-29b, miR-141, miR-200a, miR-155, miR-223, miR-326, miR-448, miR-15a/16-1, miR-15b, miR-20b, miR-21, miR-590, miR let-7e, and miR let-7i (Chen et al., 2018; Ntranos et al., 2019). Taken together, these results suggest that MS/EAE development is significantly affected by the interactions between certain miRNAs and gut microbiota.

## Microbiota-Targeted Therapies for MS

Many modern holistic approaches for health care focus on promoting beneficial gut microbiota (Gibson et al., 2017). Microbiota-targeted treatments include dietary modifications, fecal microbiota transplants (FMTs) and administration of probiotics and prebiotics. However, as with many therapies, microbiota-targeted treatments are not effective in all individuals and can also cause unintentional adverse effects, which must be minimized. Therefore, further studies are warranted to develop treatments with fewer adverse effects.

### Dietary Modifications

**FIGURE 3:**
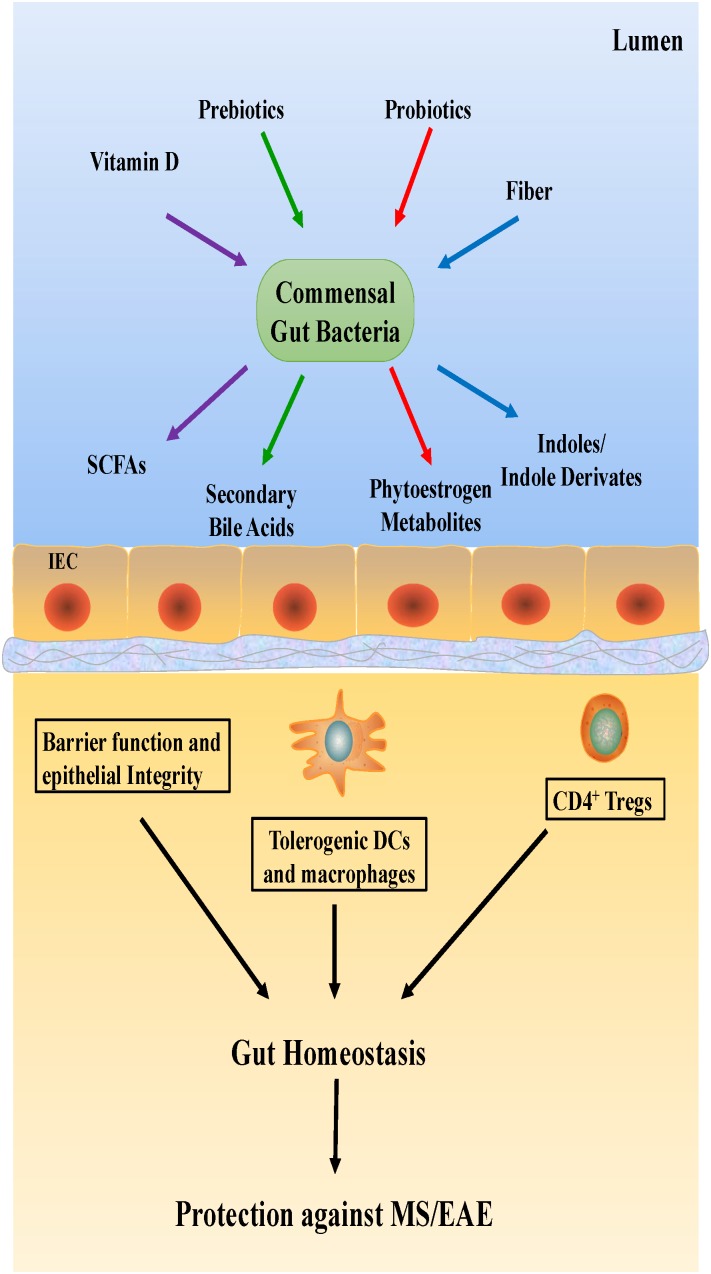
Potential mechanisms underlying dietary effects on the host immune system.

Dietary habits could be the primary determinant of gut microbial composition and function (Tilg and Moschen, 2015; Martinez et al., 2016), consequently shaping the microbial structure (Figure 3). In general, hypercaloric, high-animal fat Western diets may accelerate anabolism, alter gut microbiota composition and result in intestinal dysbiosis. Conversely, a vegetarian diet rich in fiber promotes gut eubiosis (Riccio and Rossano, 2018). Mice with high-body fat percentages that were fed a typical Western diet consisting of high levels of salt, saturated fat, protein, sugar and calories showed increased EAE severity (Hucke et al., 2016; Hammer et al., 2017); the gut flora in the mice was altered, with a notable increase in the levels of free pro-inflammatory fatty acids in the plasma. This finding suggests that Western diets reduce SCFA production by desirable gut microbiota. This reduction in SCFAs, which facilitate the production of protective Treg cells, could thus increase the incidence of autoimmune diseases (Haghikia et al., 2015).

In contrast, other studies have shown that low-calorie diets comprising high levels of fruits, vegetables and fish promote beneficial gut microbiota and reduce inflammation in the body (Riccio and Rossano, 2018). Diets that are effective at preventing EAE, reducing inflammation and increasing neuroprotection include the ketogenic diet, intermittent fasting and calorie-restricted diets. In a recent study, calorie-restricted diet was found to be more effective in reducing the symptoms of EAE than ketogenic diet (Dupree and Feinstein, 2018; Kap et al., 2018). However, this result has not yet been confirmed, and further studies to investigate the different effects of specific diets are ongoing.

Studies on MS should not be limited to studying the abundance of gut microbiota alone. Vitamin D, a nutrient that promotes Treg cell differentiation and mediates gut microbiome balance, should also be examined. Reportedly, vitamin D levels in gut microbiota vary in different diseases, such as MS (Riccio and Rossano, 2018). Similarly, a close relationship between dietary tryptophan and EAE severity in mice has been suggested, which needs to be confirmed in a future study.

### Probiotics

The use of probiotics, which produce essential vitamins and cofactors not naturally produced by the host, is a popular treatment with several health benefits, such as improvement of the immune system and inhibition of non-commensal microbiota growth (Li P. et al., 2017). The administration of probiotics is also thought to reduce the symptoms of CNS diseases. For instance, in one study, the oral administration of *Lactobacillus paracasei* and two strains of *L. plantarum* was effective in preventing EAE development in mice (Li P. et al., 2017). However, very few studies have evaluated the effects of probiotics on MS patients.

### Prebiotics and Polyphenols

Prebiotic substances also have a significant effect on microbiota. Foods with a prebiotic effect include colorful fruits, cocoa and tea leaves. Several fermentable carbohydrates also have prebiotic properties, but two oligosaccharides, fructans, and galactans, are known to be the most beneficial for *Bifidobacterium* growth. A high-fiber diet is commonly recommended due to its health benefits (Liu et al., 2015). Its protective effects in MS/EAE are associated with the ability of gut microbiota to produce SCFAs by fermenting dietary fiber. Some reports have suggested that circulating butyrate directly alters CNS function (Li W. et al., 2017; Zhang et al., 2018). Braniste et al. showed that the BBB permeability was significantly increased in germ-free mice, but after administering butyrate-producing bacteria, *Clostridium tyrobutyricum*, or an oral gavage of sodium butyrate, the BBB permeability was restored to the level present in pathogen-free mice (Braniste et al., 2014).

Similarly, another study suggested that a diet rich in polyphenols can protect against EAE (Miyake et al., 2006). In this study, mice with EAE were treated with a polyphenol extracted from Jatoba, a medicinal plant found in South America, and the polyphenol extract was found to facilitate the suppression of Th1 immunity. These results suggest that MS patients can benefit from more dietary polyphenols in addition to probiotics and prebiotics.

### FMT and Stool Substitute Transplant Therapy

Fecal microbiota transplant involves the replacement of the entire host gut microbiome to restore the desirable microbial balance. It is being increasingly used to combat a range of diseases. Benefits of FMT have been reported in three MS patients, and another study investigated how FMT affects the symptoms of secondary progressive MS (Makkawi et al., 2018). Stool substitute transplant therapy (SSTT) is similar to FMT. More research is required to determine the effects of FMT and SSTT on autoimmune diseases, such as MS.

### Other Therapies

Broad-spectrum antibiotics have been used to modify gut microbiota and treat EAE by delaying EAE development. Antibiotics can reduce the effect of EAE by modifying the T cell population in the gut assisted lymphoid tissue and nearby lymphoid tissues. For each unit of increase in IL-10 production, a simultaneous increase in Foxp3+ Treg cell production has been demonstrated (Nakamura et al., 2016; Shi and Mu, 2017).

A new alternative therapy uses helminths, which are eukaryotic worms, to combat auto-immune diseases. Although there is evidence of success in treating EAE using helminths, there is limited evidence to support the use of this therapy in MS patients.

## Perspectives

The theory that the intestinal microbiota plays a crucial role in regulating the gut–brain axis, influencing disease development and maintaining human health is gaining support. However, understanding the detailed effects of the gut microbiota in relation to MS pathophysiology requires more research. In particular, little knowledge is available regarding the mechanisms implicating the gut microbiota in MS onset and progression. These mechanisms could be elucidated by studying the gut microbiota-mediated miRNA–MS/EAE axis.

As an increasing number of studies confirm the association between gut microbiota and neuroimmune inflammatory diseases, treatment involving alteration of gut microbiota will become more appealing. However, the widespread application of such treatments may not happen soon as there is limited evidence of their potential beneficial effects in humans. Thus, more large-scale trials focusing on identifying the microbes that are integral to MS development and understanding how they function are warranted. The results of such studies will assist in developing effective treatments to prevent MS by altering the gut microbiome.

## Author Contributions

YF finished the first draft of the manuscript. JZ critically revised the manuscript. Both authors approved the submission of the manuscript.

## Conflict of Interest Statement

The authors declare that the research was conducted in the absence of any commercial or financial relationships that could be construed as a potential conflict of interest.
